# UV Irradiance and Albedo at Union Glacier Camp (Antarctica): A Case Study

**DOI:** 10.1371/journal.pone.0090705

**Published:** 2014-03-05

**Authors:** Raul R. Cordero, Alessandro Damiani, Jorge Ferrer, Jose Jorquera, Mario Tobar, Fernando Labbe, Jorge Carrasco, David Laroze

**Affiliations:** 1 Departamento de Física, Universidad de Santiago de Chile, Santiago, Chile; 2 Escuela Superior Politécnica del Litoral, Guayaquil, Ecuador; 3 Departamento de Ingeniería Mecánica, Universidad Técnica Federico Santa María, Valparaíso, Chile; 4 Dirección Meteorológica de Chile, Santiago, Chile; 5 Instituto de Alta Investigación, Universidad de Tarapacá, Arica, Chile; University of Oxford, United Kingdom

## Abstract

We report on the first spectral measurements of ultraviolet (UV) irradiance and the albedo at a Camp located in the southern Ellsworth Mountains on the broad expanse of Union Glacier (700 m altitude, 79° 46′ S; 82° 52′W); about 1,000 km from the South Pole. The measurements were carried out by using a double monochromator-based spectroradiometer during a campaign (in December 2012) meant to weight up the effect of the local albedo on the UV irradiance. We found that the albedo measured at noon was about 0.95 in the UV and the visible part of the spectrum. This high surface reflectivity led to enhancements in the UV index under cloudless conditions of about 50% in comparison with snow free surfaces. Spectral measurements carried out elsewhere as well as estimates retrieved from the Ozone Monitoring Instrument (OMI) were used for further comparisons.

## Introduction

In general, surface UV climate is determined by the total ozone column, cloudiness, ground reflectivity (i.e. the albedo), and local aerosols. Although the latter may significantly modulate the surface UV, in Antarctica the load of aerosols is extremely low [1]. Moreover, while heavily overcast conditions can reduce surface UV irradiance up to 90% in the Antarctic Peninsula [2],[3], the role of clouds is less important on the Antarctic plateau. Therefore, surface UV in Antarctica is driven by ozone and by albedo. The UV climatology at several Antarctic sites has been already studied [2]–[6].

On the Antarctic plateau, large seasonal ozone losses (which occur every year in the late August to early October period [7]) lead to a significant increase in surface UV radiation. Ground-based measurements have shown that the average spring erythemal irradiance for 1990–2006 is up to 85% greater than the modeled irradiance for 1963–1980 [8].

The high albedo of a snow-covered surface has a large effect on the global UV radiation, due to multiple reflections between the ground and the scattering atmosphere [9]. Model calculations of UV irradiance for cloudless sky show enhancements in irradiance levels of nearly 50% at 320 nm for a snow-covered surface, in comparison with snow-free surfaces [10]. Snow albedo depends on snow characteristics (grain size, age of snow, snow height, and soot on the snow) and on other factors such as the solar zenith angle (SZA), atmospheric parameters, geometric pattern of the snow surface, and morphology of the area surrounding the measurement site [11].

So far, the ozone depletion in Antarctica has been the dominant factor in the alterations observed in the UV irradiance. However, as a consequence of climate change, the albedo (that is the other major parameter determining the surface UV) is likely to change in its absolute amount, in its qualitative structure or in its temporal pattern.

The consequences of changes in the Antarctic albedo are beyond local variations in the UV. The surface energy budget of the Antarctic continent is significantly dependent on the surface albedo. Therefore a change in prevailing climatic conditions (triggered for example by a change in temperature) can enhance feedback mechanisms. The so-called albedo feedback is a mechanism that enhances climate change, particularly in regions with snow and ice cover [12]. As the Earth warms, the surface reflects less shortwave radiation to space due to changes in the coverage and reflectivity of the surface ice cover, which leads to additional warming [13]. If temperature increases in Polar Regions we can expect both a decrease of the surface albedo in some areas (due to ice melting) and an enhancement in other areas (due to the freshly fallen snow caused by increased precipitations) [14]. More snow and ice melting, more water vapor and in turn more clouds may lead to changes in the Earth’s albedo and alterations in the global energy budget. Indeed, general circulation models (GCMs) [15]–[17] have shown a high sensitivity to changes in the Earth’s albedo [16].

Since albedo significantly affects the UV climatology in Polar Regions (and also plays an important role in the Earth’s climate warming pattern), its characterization is required. However, quality-controlled measurements of the albedo in Polar Regions are sparse and few spectral measurements have been reported [9],[18]–[23].

Prior measurements at Amundsen-Scott (South Pole) Station and at Vostok Station found that the albedo was nearly independent of snow grain size and ranged from 0.96 to 0.98 in the UV and the visible part of the spectrum [18]; it has been also observed that the albedo slightly increased with the SZA (which agrees with prior results [24]).

Measurements in Sodankylä (67°22′N, 26°39′E, 179 m altitude) [21], and at Neumayer station (70°39′S, 8°15′W) [9], detected that the albedo decreased (up to 10%) as the SZA decreased through the day. The reductions range from 0.77 to 0.67 in the Arctic, and from 0.96 to 0.86 in the Antarctica [25]. The changes in the Antarctic albedo essentially agree with measurements carried out by using broadband instruments at Hells Gate Station (74°51′S, 163°48′E, 20 m altitude), at Neumayer Station, and at Dome Concordia Station (75°09′S, 123°06′E, 3232 m altitude) [11]. Snow metamorphism, sublimation during the day, and refreezing and/or crystal formation/precipitation during the night can explain the observed trends [11]. The spectral albedo of melting snow has been also measured near Barrow (Alaska) [19], near Davis Station (68°34′S, 77°58′E) [20] and in Sodankylä [22].

In this paper, we report on the first quality-controlled spectral measurements of the UV irradiance and the albedo at Union Glacier Camp, located in the southern Ellsworth Mountains on the broad expanse of Union Glacier; 3,030 km from the southern tip of Chile and about 1,140 km from the South Pole (700 m altitude, 79° 46′ S; 82° 52′W). The mean ice thickness of Union Glacier is 1450 m (with a deep subglacial topography; about 900 m below sea level [23]). The measurements were carried out by using the so-called USACH spectroradiometer from the Universidad de Santiago de Chile (USACH, Chile) during a campaign (in December 2012) meant to weight up the effect of the local albedo on the UV irradiance.

## Materials And Methods

### 1. Ground-Based Measurements

Quality-controlled measurements of the surface UV require double monochromator-based spectroradiometers. Instruments developed according to the specifications defined by the World Meteorological Organization (WMO) [26] and the Network for the Detection of Atmospheric Composition Change (NDACC) [27] can produce a radiometric stability better than 1% [28].

The accuracy of NDACC-certified instruments has been tested by intercomparison Campaigns [29],[30],[31]. The differences between NDACC-certified instruments are normally within the bounds defined by the involved uncertainties; up to 4% for UVA wavelengths and up to 10% for UVB wavelengths [32]. By comparison, for broadband instruments (calibrated by using spectroradiometers) uncertainties in the range 7%–16% have been reported [33],[34]).

The UV Index (UVI) can be retrieved from spectral measurements by integrating the UV spectra (weighted by the Erythema action spectrum [35]). The uncertainty of UVI values computed from measurements by NDACC-certified instruments has been estimated to be about 5% [36]. Uncertainties of erythemal daily doses (computed also from spectral measurements) are expected to be similar.

Quality-controlled UV time series are limited. In Antarctica, the spectral UV irradiance has been monitored for almost two decades by the U.S. National Science Foundation [8]. Based on those time series, the UV climatology at several Antarctic locations (Palmer Station, McMurdo station and at South Pole station) has been reported [2],[4],[5].

Spectral measurements reported below were carried out by using the USACH spectroradiometer, which is based on a double monochromator Bentham DMc150F-U, 150 mm focal length, and 1800 lines/mm gratings, fitted with a photomultiplier (PMT) as detector. The Full With at Half Maximum (FWHM) of the USACH spectroradiometer is 2 nm. The system is operated within a temperature-controlled weatherproof box. An input optics for global irradiance fitted with a flat diffuser was used. Although it is not a NDACC-certified instrument, our spectroradiometer complies with NDACC specifications [27] and the WMO recommendations [26]. The USACH spectroradiometer sampled the irradiance every 1 nm (in the range 280–400 nm); scans were carried at a 30 min interval. The absolute calibration of the system was achieved by using a field calibrator fitted with a baffled 100 W quartz halogen lamp. In addition to in situ calibration, quality assurance included corrections for dark signal and for cosine error of the diffuser (see [26] for details). Based on the certificate of the lamp and the transfer of calibrations, we estimated the uncertainty involved in the absolute calibration to be up to 4% for UVA wavelengths and up to 10% for UVB wavelengths (see [32] for details).

### 2. Satellite Estimates

Satellite-derived estimates of the total ozone column (TOC) data have been compared with ground-based measurements [37],[38]. However, under specific conditions (e.g., high satellite SZA, high surface albedo) and/or for Polar Regions (e.g., at high latitudes), a poorer agreement has been reported [39].

Satellite-derived estimates of the surface UV are normally derived from other products (e.g., ozone, albedo, aerosols and cloud cover) by using radiative models and then uncertainties tend to be somewhat higher especially for partly cloudy and overcast conditions [40],[41], and over snow-covered surfaces [42],[43].

Over polluted areas, validations of OMI-derived data have generally reported that the UV estimates are biased high [44]–[52]. Among the factors that explain the overestimation have been pointed out the limited spatial resolution [46],[51], the lack of sensitivity of OMI to the boundary layer [39],[41],[44],[45],[48],[52], and the effect of high SZA on the nadir-viewing instruments [39. However, over snow-covered surfaces the OMI-derived dose is generally lower than the ground-based measurement because the OMI algorithm uses climatological surface albedo that may then be lower than the actual effective surface albedo [42],[43]. Part of the problem is that a portion of the observed reflectivity may be incorrectly interpreted as cloud cover, which reduces the estimated irradiance [3].

As shown below, in this study our ground-based measurements were compared with OMI data. The UV estimates were retrieved from OMUVB products (version 1.3). We selected overpass OMI data minimizing the distance between the ground station and the center of the satellite pixel and, due to the presence of the mountains, discarding pixels at altitudes greater than 1000 m asl. The ozone estimates shown below over the period 2004–2012 were retrieved from OMTO3 products.

### 3. Radiative Transfer Models

Surface UV can also be computed by using radiative transfer models from a set of parameters that include the albedo, the total ozone column, the SZA, as well as the characteristics of aerosols and clouds. Under cloudless conditions and for UVB wavelengths the uncertainties of model products can be and up to 20% for sites with very large aerosol load up, and up to 9% for unpolluted sites [53],[54].

The reflectivity of the areas with spatial variation in the albedo [55],[56] is often characterized by using the “effective” albedo (the albedo that when used as input into a model reproduces the measured spectrum) [57],[58].

Comparisons with ground-based measurements have validated model products [59],[60] under cloudless conditions. Although the characterization of the cloud effect is still difficult, models are useful for checking the consistency of surface measurements [2]. In this work, as radiative transfer model we used UVSPEC [61]. The selected radiative transfer solver was the DISORT solver [62]; the extraterrestrial spectrum was quoted from Gueymard [63].

### 4. Meteorology At Union Glacier Camp

In general, the meteorology at the Union Glacier Camp is defined by the high pressure centered in the interior of the continent, the orography of the region, and the fact that it is located a distance of ∼750 km from the edge of Ronne Ice Shelf and the coast line of Ellsworth Land. An automatic Weather Station (AWS) deployed by the Antarctic Logistic & Expeditions (ALE) Company, nearby the Union Glacier runway (79°46 S, 83°16 W) in early February 2010, provides meteorological data to characterize the area. According with the almost 3 years of data, the near-surface air temperature shows a “winter coreless” behavior reaching around −26 to −28°C from April to September and around −6°C in January. The average relative humidity after temperature correction for freezing environment [64] is ∼77%; while, the prevailing wind direction year-round is from the South-South west (190–200° magnetic direction) with an average wind speed of ∼20 ms^−1^, revealing the katabatic origin of the wind field [65] affecting the area. On the other hand, the annual cloud cover fraction, according with recent results of the joint CloudSat-CALIPSO satellite data [66] over the southern Ellsworth Mountains region, can range between 30–40% in summer and 60–70% in winter.

## Results And Discussion

### 1. Irradiance Measurements

Despite the fact that no specific permissions are required for the locations/activities reported in our paper, the Chilean Antarctic Institute (INACH, www.inach.cl) issued a permission after our request for measurements that we carried out at an installation in Antarctica (at Union Glacier Camp) under their supervision. Moreover, we confirm that the field studies did not involve endangered or protected species.

**Figure 1:**
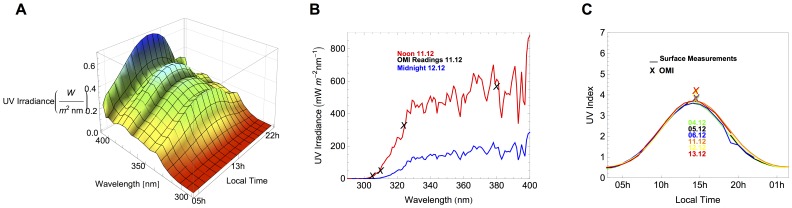
UV Irradiance at Union Glacier Camp. a) Ground-based measurements of the UV irradiance on 04.12.2012 (cloudless conditions). b) Spectra measured at noon on 11.12.2012 (cloudless conditions, SZA = 57°, red line) and at midnight on 12.12.2012 (cloudless conditions, SZA = 77°, blue line). Crosses stand for OMI readings at 305 nm, at 310 nm, at 324 nm and at 380 nm. c) Lines stand for UVI computed from ground-based spectral measurements on 04.12.2012 (green line), on 05.12.2012 (black line), on 06.12.2012 (blue line), on 11.12.2012 (orange line), on 12.12.12 (yellow line), on 13.12.2012 (red line). Crosses stand for OMI estimates of UVI.

Figure 1a shows the spectral measurements carried out on 04.12.2012 (day.month.year) under cloudless conditions. Figure 1b depicts the spectra measured on 11.12.2012, also under cloudless conditions at noon (SZA = 57°) and at midnight (SZA = 77°). Figure 1c shows the UVI computed from our spectral measurements for 4-13.12.2012. The predominant cloudless conditions led to the smooth curves shown in figure 1c.

Figures 1b and 1c also show some satellite estimates. Crosses in figure 1b stand for OMI readings at 305 nm, at 310 nm, at 324 nm and 380 nm. OMI-derived estimates of UVI are also depicted by crosses in figure 1c. OMI measures the solar reflected and backscattered radiation in the wavelength range from 270 nm to 500 nm, that are used to retrieve the TOC, aerosol and cloud cover characteristics, surface UV irradiance and gas traces. The ground pixel size at nadir position is 13×24 km (along × across track) for total ozone column.

Despite the different spectral resolution (OMI resolution is 0.55 nm in the ultraviolet and 0.63 nm in the visible while the FWHM of our instrument was 2 nm), figure 1b shows a reasonable good agreement between our measurements and the OMI readings at 305 nm, at 310 nm, at 324 nm and 380 nm. Without correcting the effect of the resolution, the differences were within the range ±10% at 305 nm, at 324 nm and 380 nm; differences up to 30% were found at 310 nm.

In figure 1c, it can be observed that the UVI peaked at 3.8 on 13.12.2012; this value is about 10% lower than the corresponding UVI estimate retrieved from OMI. Also on 13.12.2012, the difference between the Erythemal Daily Dose (EDD) computed from our ground-based measurements (4113 J/m2), and the corresponding OMI-derived estimate (4448 J/m2), was about 8%. The OMI estimates of the EDD during the Campaign ranged from 4000 J/m2 on 11.12.2012 to 4448 J/m2 on 13.12.2012. When comparing ground-based estimates and satellite-derived data, we found during the campaign differences that ranged from 2% to 10% (in the case of the UVI) and from 0.1% to 8% (in the case of the EDD). These differences were anyway always within the expanded uncertainty bounds of our measurements [36].

**Figure 2:**
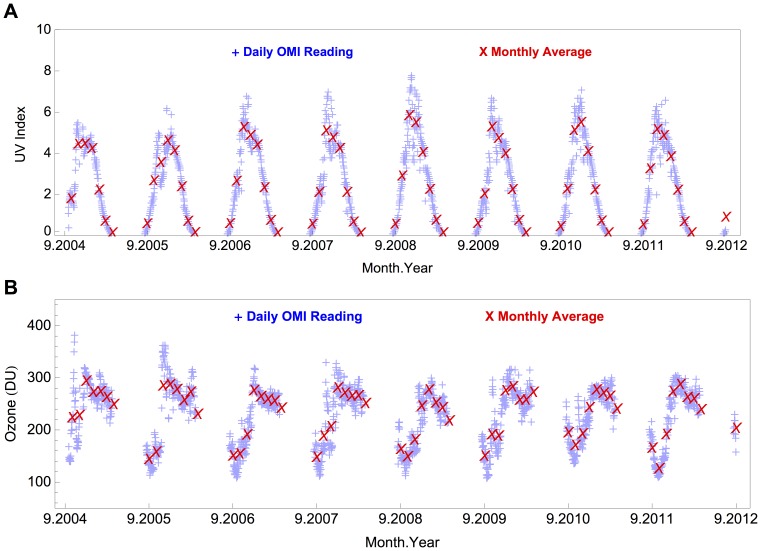
Time series of OMI-derived data at Union Glacier Camp.Figure 1. UV Irradiance at Union Glacier Camp. The number “9” in the abscise tick labels means “September”. The blue crosses stand for the daily estimates; the red crosses stand for the monthly averages of the OMI-derived data. (a) Noontime UV index (UVI). (b) Total ozone column.

Figure 2a shows the time series of the OMI-derived estimates of UVI at noon at Union Glacier Camp. Due to the high SZA, OMI readings are not available for the period May–August. As shown in figure 2a, annually in spring, the noon­time UVI is typically lower than 6 (although UVI estimates higher than 8 have been retrieved due to the seasonal ozone depletion). As shown in figure 2a, the average of UVI estimates computed for November 2008 was about 20% greater than the average for the same month over the 4 preceding years.

Figure 2b shows the ozone estimates over the period 2004–2012 retrieved from OMTO3 products. Large seasonal ozone losses are apparent in figure 2b and values close to 100 DU have been recorded every year in October [67]. By comparing figures 2a and 2b, becomes clear that the seasonal ozone depletion leads to significant increments in the UVI and in its variability. In this work, the monthly average and the corresponding variability were taken as average and the standard deviation, respectively, of the daily estimates. Table 1 shows the monthly average and the corresponding variability of OMI products. The average of the daily ozone estimates for September (158 DU) is typically about 40% lower than for January (275 DU). The variability of ozone data ranges between 5% in January and 26% in November (see Table 1). In the case of UV products, the variability of the “clear-sky UVI” computed for October (34%) is roughly 3 times greater than for January (11%).

**Table 1:** **Table 1.** UV Climatology at Union Glacier Camp computed by using the OMI-derived data over the period 2004–2012.

		Jan	Feb	March	April	Sept	Oct	Nov	Dec
Ozone [DU]	**Average**	**275**	**261**	**259**	**241**	**158**	**161**	**203**	**267**
	Variability	13	16	20	18	28	40	53	30
	Variability [%]	5	6	8	7	18	25	26	11
UVI (Clear-Sky)	**Average**	**4.14**	**2.27**	**0.70**	**0.11**	**0.53**	**2.59**	**5.17**	**5.03**
	Variability	0.46	0.61	0.34	0.03	0.40	0.89	1.17	0.74
	Variability [%]	11	27	49	30	77	34	22	14
UVI	**Average**	**4.09**	**2.22**	**0.68**	**0.11**	**0.53**	**2.52**	**5.02**	**4.95**
	Variability	0.53	0.64	0.35	0.03	0.40	0.90	1.22	0.78
	Variability [%]	13	29	51	30	77	36	24	16
Erythemal	**Average**	**3825**	**1691**	**425**	**38**	**278**	**1767**	**4058**	**4849**
Daily Dose	Variability	756	657	249	23	245	799	889	782
(J/mˆ2)	Variability [%]	20	39	58	59	88	45	22	16
Surface Albedo	**Average**	**0.89**	**0.87**	**0.83**	**0.78**	**0.76**	**0.85**	**0.88**	**0.89**

The variability was taken as being equal to the standard deviation of the OMI-derived values.

doi:10.1371/journal.pone.0090705.t001

**Figure 3:**
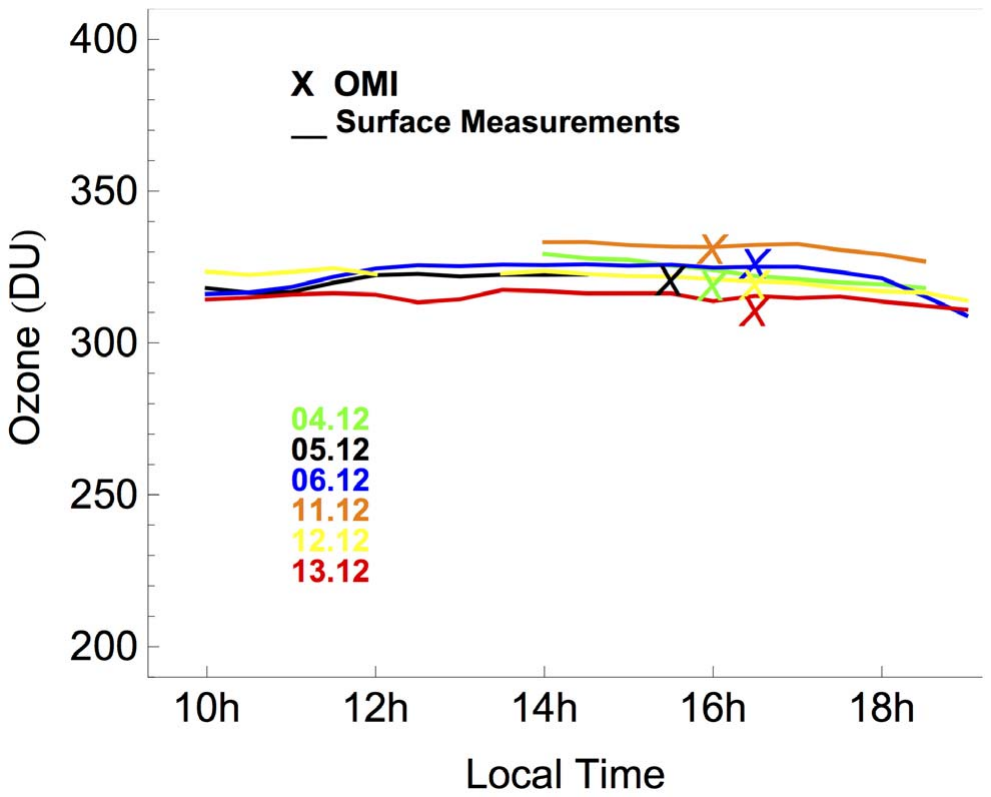
Ozone Column at Union Glacier Camp. Lines stand for total ozone column retrieved from ground-based measurements. Crosses stand for ozone estimates retrieved from OMI. Color indicates the dates: 04.12.2012 (Green); 05.12.2012 (black); 06.12.2012 (blue); 11.12.2012 (orange), 12.12.2012 (yellow), 13.12.2012 (red).

We compared the ozone estimates retrieved from OMI, with the ozone computed from our measurements of the UV spectra carried out in December 2012. The ozone was retrieved from our measurements of the global irradiance by applying a method that implied comparing the ratio (between irradiances measured at different wavelengths) with a synthetic chart of this ratio computed for a variety of ozone values [68]. Figure 3 shows the total ozone column progression for 4-13.12.2012 computed from our ground-based measurements. As shown in the figure, our daily measurements agree with OMI-derived estimates of ozone (see crosses in Figure 3). Although the detected differences were slight (ranging from 0.1% to 3% (i.e. up to about 10 DU)). due to the high sensitivity of the UVI to the ozone, they can explain most of the differences in the UVI estimates shown in figure 1c. Estimations of the total ozone column retrieved from spectral measurements at other Antarctic locations have been also found to agree with TOMS data [2],[5],[69].

We further exploited our ground-based measurements studying the possibility of an unlikely aerosol modulation. The sky in the Antarctic interior is breathtakingly clear: there is no source of dust or other particulate matter but no measurements of the properties of aerosol loading at Union glacier Camp have been reported. We carried a limited number of spectral measurements of both the global and the diffuse irradiance (by using a shadow ring) at Union Glacier Camp around noon on 09.12.2012. Both, the AOD and the single scattering albedo (SSA) were retrieved from these quality-controlled spectral measurements by applying methods based on the comparison of the measured spectral irradiance with UV spectra computed by using a radiative transfer model [70]–[72]. According to these methods, the retrieved values of the AOD and SSA are those leading to the best match between the measured and the computed spectra [70]–[72]. By using the methods described above, we estimated that the AOD and the SSA at Union Glacier Camp are about 0.02 and 0.99, respectively. These values stand for an extremely low aerosol loading. Actually, due to the uncertainty associated with our aerosol estimates, assuming the total lack of aerosols in the area is also reasonable [9]. At Neumayer, extremely low aerosol loading has been recently measured [1].

Since our measurements were carried our under cloudless conditions, they did not allow us to weigh up the effect of local clouds on the UV irradiance. However, the effect of the high albedo of the surrounding snow-covered surface was assessed (see sections 4 and 5).

### 2. Albedo Measurements

The albedo is normally taken as being equal to the ratio between the up- and downwelling global radiation. Although measurements of the broadband albedo are normally carried out by using two radiometers [26], our spectral measurements were carried out (at 300–800 nm wavelength range) by using a single spectroradiometer and a rotating input optics that was set up at about 2.5 m above the snow surface. This means that the albedo assessments required measuring sequentially the up- and downwelling global radiation. Due to the time needed to accomplish a scan, the up- and downwelling spectra were separated in time about 5 min. Pairs of scans were carried out at 30 min intervals. A similar method based on the rotation of the input optics has been previously used in Antarctica [9]. Since the measurements of the up- and downwelling radiation were not carried out at the same time (i.e. at the same SZA), the spectra measured at 30 min intervals were linearly interpolated. Then, we estimated the up- and downwelling radiation for certain common SZAs.

**Figure 4:**
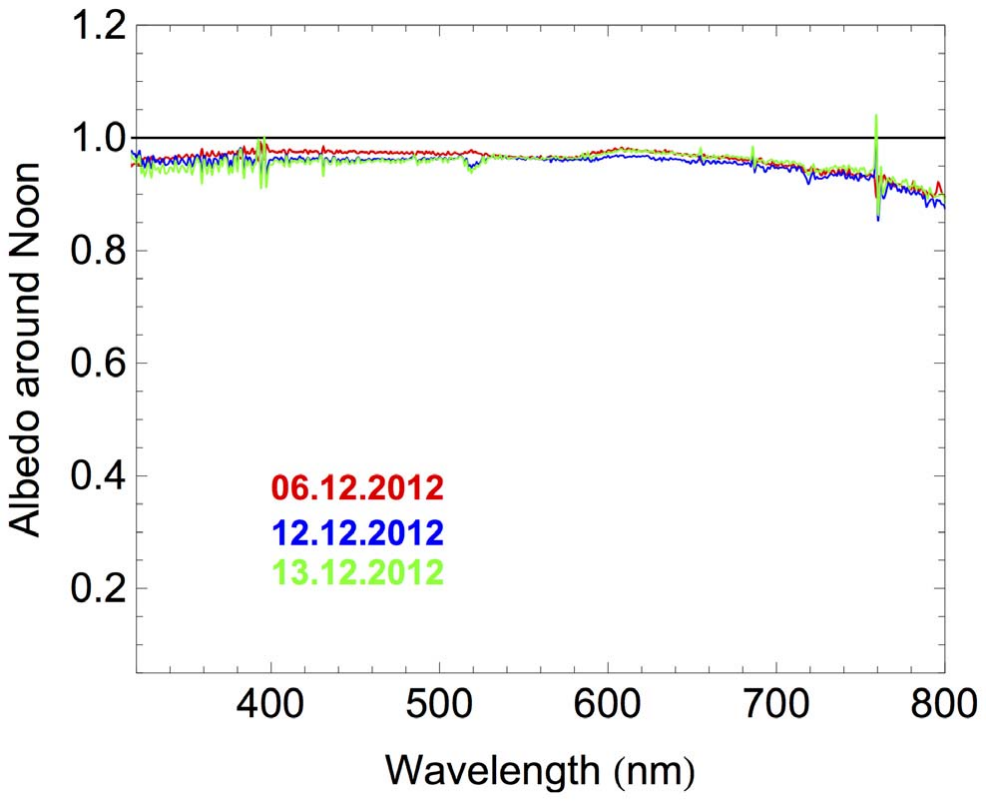
Spectral albedo measured under cloudless conditions around noon at Union Glacier Camp. Color indicates the dates: 06.12.2012 (Red); 12.12.2012 (Blue); 13.12.2012 (Green). Note that the narrow spectral variations in figure 4a have no physical meaning and are due to insignificant misalignments in the grating of our scanning double monochromator when measuring sequentially the up- and downwelling global radiation.

Figure 4 shows the spectral albedo measured around noon. No significant differences were observed between measurements carried out on different days around noon under cloudless conditions. As shown in figure 4, the spectral albedo around noon was nearly constant in the UV and visible range but decreased at wavelengths longer than 600 nm; the lowest albedo (about 0.86) was measured at 800 nm. The diminishment of the albedo with the wavelength in the near infrared was expected since it has been reported by all prior spectral measurements of the albedo over snow-covered surfaces [9],[18]–[23].

Note that the albedo measurements shown in figure 4 are slightly lower than the values measured at Neumeyer station [9]. The difference maybe linked with characteristic of the snow, which is ultimately determined by the local meteorology. While at Neumeyer station the albedo of fresh snow was often measured, our measurements were carried out over a glacier covered by 2 m of old snow with grain radio of about 1 mm; old coarse-grained has lower albedo than fresh snow [20].

Measurements through the day have detected that the albedo decreased (up to 10%) as the SZA decreased through the day [9],[21]. These diminishments range from 0.77 to 0.67 in the Arctic, and from 0.96 to 0.86 in the Antarctica [25]. The change in the albedo through the day has been attributed to changes in the snow characteristics [11]. Moreover, snow melting during daytime and refreezing during night may lead to albedo asymmetries (i.e. differences in the albedo measured at the same SZA, at different times of day) [25]. However, we did not detect significant chances in the albedo through the day at Union Glacier Camp. We attributed the apparent albedo stability to the relatively small variations through the day (from −12°C to −9°C) in the air temperature logged by the ALE’s AWS during our albedo measurements.

Note that our measurements were carried out about 3 km away from the closest peak (1450 m altitude). Therefore, the slopes of the surrounding mountains were in the field of view of the input optics. However, the complex topography surrounding Union Glacier Camp did not lead to any measurable effect on our ground-based measurements. Indeed, we checked the possibility of asymmetries (i.e. differences between spectral measurements carried out at the same SZA, at different times of day). We found that the differences were always within the uncertainty bounds of our measurements [32].

**Figure 5:**
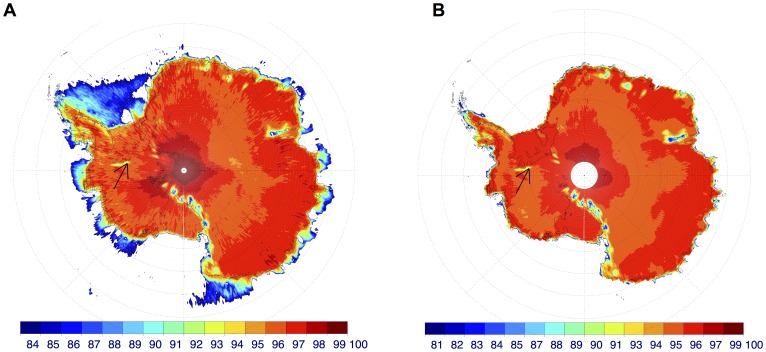
Satellite Albedo. a) Climatology albedo from OMI surface reflectance data (LER) at 354 nm for December. Arrow indicates the location of the Union Glacier Camp. b) Climatology albedo from OMI reflectance data (LER) at 354 nm for snow-covered scenes. Arrow indicates the location of the Union Glacier Camp. See the text for details.

The surrounding topography did affect satellite data. Figure 5a depicts the surface reflectance map of Antarctica from OMI Lambertian equivalent reflectivity (LER) at 354 nm. LER is the required reflectance of an isotropic surface needed to match the observed top of the atmosphere (TOA) reflectance in a pure Rayleigh scattering atmosphere [73]. Therefore, it can be used to roughly estimate the actual surface albedo. Figure 5b shows the average of the surface reflectance data at 354 nm computed for December by using the data over the period 2005–2009 [73]. This is the albedo used by OMI algorithm to compute the UV surface irradiance (see Table 1). As expected the general pattern shows a very high surface albedo over almost all the Antarctic continent (with values roughly equal to or greater than 0.95). Lower reflectivities can be observed over the Transantarctic Mountains (close to the Ross Ice Shelf) and over the Ellsworth Mountains. Due to the katabatic winds the Antarctic mountain slopes are often snow-free and showing their underlying dark bedrock. Considering the relatively large OMI pixel (maps in figures 5a and 5b have a 0.5×0.5 degree resolution), the difference between ground-based measurement of albedo (about 0.95 in the UV and visible) and the lower reflectivity recorded by satellite (about 0.9 at 354 nm, see Table 1) may be partly explained by these small dark areas inside satellite bright pixels and by the Lambertian approximation.

Figure 5b shows the annual average of the reflectance data at 354 nm of snow-covered scenes (with relaxed cloud-screening criteria [74]). In contrast to figure 5a, clear-sky OMI scenes have been selected by using cloud and aerosol data from the MODIS/Aqua satellite instrument that flies 12 min ahead of OMI. The result is a reflectivity dataset that does not rely on statistical methods to eliminate cloud effect [74]. Despite some minor differences in absolute values (likely related to of the different periods of time considered in the datasets), the patterns are nearly the same.

### 3. Albedo Effect On Uv

**Figure 6:**
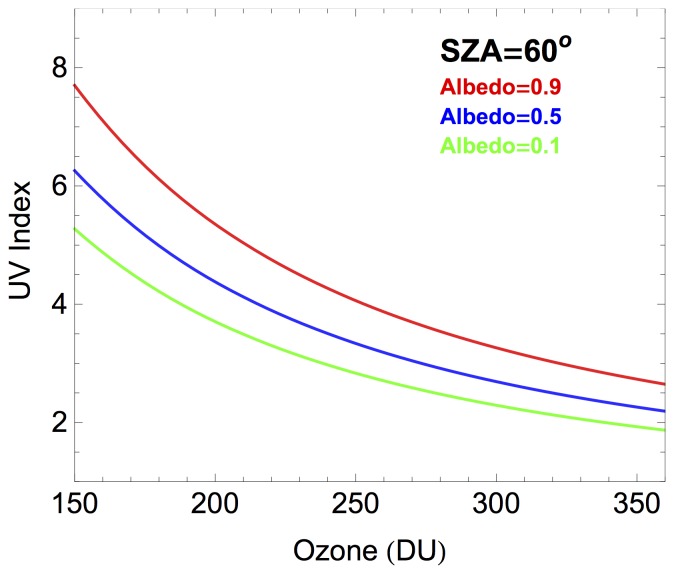
Change in the UVI with the albedo. The UVI was computed by using the UVSPEC model under the conditions observed at Union Glacier Camp at 11∶40 LT on 06.12.2012 (SZA = 60°, AOD = 0.02; SSA = 0.99; altitude = 700 m) but assuming different ozone column values as well as different albedo levels (Red line: 0.9; Blue line: 0.5; Green line: 0.1).

In order to weight up the effect of the albedo, we computed the UVI by using the UVSPEC model under the conditions observed at Union Glacier Camp at 11∶40 LT on 06.12.2012, 2012 (SZA = 60°, AOD = 0.02; SSA = 0.99; altitude = 700 m; cloudless conditions) but assuming different ozone values as well as different albedo levels. As pointed out above, as radiative transfer solver was used the DISORT solver [69]; the extraterrestrial spectrum was quoted from Gueymard [68]. Figure 6 shows that despite of the ozone, a surface reflectivity greater than 0.9 in the UV (as that measured at Union Glacier Camp) led to enhancements in the UVI under cloudless conditions of about 50% in comparison with snow free surfaces (albedo = 0.1).

The effect of the albedo can be also weighted up by comparing our ground-based measurements at Union Glacier Camp with other spectra measured at snow free locations (albedo = 0.1). We used some of our measurements carried out in the austral summer at Paranal Observatory (2,635 m altitude, 24°37′S, 70°24′W) and in Santiago de Chile (500 m altitude, 33°27′ S, 70°41′ W).

**Figure 7:**
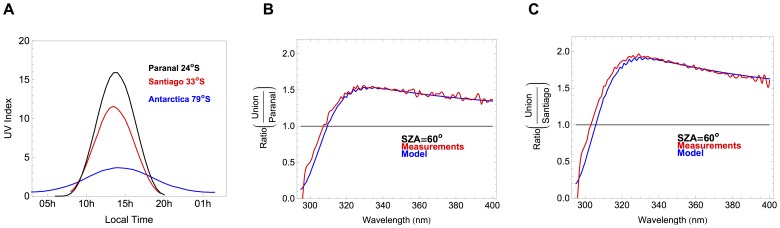
UV index at different locations. a) UV index computed from spectral measurements carried out by using the USACH spectroradiometer at Paranal Observatory on 09.01.2013 (Black line; minimum SZA = 2.6°), in Santiago de Chile on 10.12.2011 (Red line; minimum SZA = 11°), and at Union Glacier Camp on 05.12.2012 (Blue line; minimum SZA = 56.7°). b) Red line: ratio between UV spectra at SZA = 60° measured at Union Glacier Camp (on 05.12.2012) and at Paranal Observatory on (09.01.2013). Blue line: ratio between modeled UV spectra at Union Glacier Camp (albedo = 0.95; AOD = 0.02; SSA = 0.99; altitude = 700 m; ozone = 330 DU) and at Paranal Observatory (albedo = 0.1; AOD = 0.15; SSA = 0.7; altitude = 2,635 m; ozone = 260 DU). c) Red line: ratio between UV spectra at SZA = 60° measured at Union Glacier Camp (on 05.12.2012) and at USACH station in Santiago de Chile (on 10.12.2011). Blue line: ratio between modeled UV spectra at Union Glacier Camp (albedo = 0.95; AOD = 0.02; SSA = 0.99; altitude = 700 m; ozone = 330 DU) and in Santiago de Chile (albedo = 0.1; AOD = 0.3; SSA = 0.7; altitude = 500 m; ozone = 265 DU).

Figure 7a shows the UV index computed from spectral measurements carried out at Union Glacier Camp on 05.12.2012, at Paranal Observatory on 09.01.2013, and in Santiago de Chile on 10.12.2011. Mostly due to the latitude (and in turn to the SZA), peak UVI values at Union Glacier Camp are significantly lower than those in Santiago and at Paranal Observatory. The figure changes when further comparisons are carried at common SZA.

Figure 7b shows the ratio between UV spectra at SZA = 60° measured at Union Glacier Camp (on 05.12.2012) and at Paranal Observatory (on 09.01.2013). Figure 7c shows the ratio between UV spectra at SZA = 60° measured at Union Glacier Camp (on 05.12.2012) and at USACH station in Santiago de Chile (on 10.12.2011). In figures 7b and 7c we also show the ratio between modeled UV spectra computed by using the UVSPEC model under the conditions observed at the moment of the ground-based measurements (see details in the captions).

It can be seen in figures 7b that the UVA irradiance at Union Glacier Camp is significantly greater than at Paranal Observatory (the difference is 60% at 330 nm). Most of this difference is due to the albedo, and it would be even greater but the altitude of the observatory attenuates the difference. In figures 7c, it can be observed that the UVA irradiance at Union Glacier Camp is also significantly greater than in Santiago (the difference is 100% at 330 nm). Although again most of the difference is due to the albedo, the aerosol load in the case of Santiago boosts the difference. As shown in figures 7b and 7c, the differences in the UVB range are counterbalanced by the ozone (about 30% lower in Santiago and at Paranal than at Union Glacier Camp, see captions for details). However, despite the relatively high ozone, the UVI at Union Glacier Camp on 05.12.2012 at SZA = 60° (330 DU), was greater than in Santiago on 10.12.2011 at SZA = 60° (265 DU) and than at Paranal observatory on 09.01.2013 at SZA = 60° (260 DU).

## Summary And Conclusions

We report on the first quality-controlled spectral measurements of the albedo and the UV irradiance at Union Glacier Camp, located in the southern Ellsworth Mountains on the broad expanse of Union Glacier. The measurements were carried out in December 2012 by using a double monochromator-based spectroradiometer during a campaign meant to weight up the effect of local albedo on the UV irradiance.

The UVI, the TOC, the AOD and the SSA, were computed from our spectra. We found that our surface measurements roughly agreed with OMI estimates of UVI and TOC; the detected differences ranged from 0.1% to 3% (i.e. up to about 10 DU) in the case of the TOC, and from 2% to 10% in the case of the UVI. Moreover, the AOD and the SSA retrieved from our surface measurements allowed us to conclude that the aerosol loading at Union Glacier Camp is extremely low (i.e. the aerosol influence in the area is negligible).

We also found that the spectral albedo measured at noon was about 0.95 in the UV and the visible part of the spectrum. We did not detect significant asymmetries (i.e. differences in the albedo measured at the same SZA, at different times of day). The differences between albedo values sampled at different times of day were always within the uncertainty bounds of our measurements.

In order to weight up the effect of the albedo, we compared our ground-based measurements at Union Glacier Camp with other spectra measured at snow-free locations. We used some of our measurements carried out in the austral summer at Paranal Observatory (2,635 m altitude, 24°37′S–70°24′W) and in Santiago de Chile (500 m altitude, 33°27′ S–70°41′ W). Further modeling allowed us to confirm that the surface reflectivity similar to that at Union Glacier Camp around noon (albedo = 0.95) led to enhancements in the UVI under cloudless conditions of about 50% in comparison with snow free surfaces (albedo = 0.1).

## References

[1] Weller R, Minikin A, Petzold A, Wagenbach D, König-Langlo G (2013) Characterization of long-term and seasonal variations of black carbon (BC) concentrations at Neumayer, Antarctica. Atmos Chem Phys 13: 1579–1590.

[2] Bernhard G, Booth CR, Ehramjian JC (2005) UV climatology at Palmer Station, Antarctica. In: Ultraviolet Ground- and Space-based Measurements, Models, and Effects V, Bernhard G, Slusser JR, Herman JR, Gao W editors, Proceedings of SPIE, 5886, 51–62.

[3] Cordero RR, Damiani A, Seckmeyer G, Riechelmann S, Laroze D, et al (2013) Satellite-derived UV Climatology at Escudero Station (Antarctic Peninsula). Antarctic Science 25(6): 791–803.

[4] Bernhard G, Booth CR, Ehramjian JC (2004) Version 2 data of the National Science Foundation’s Ultraviolet Radiation Monitoring Network: South Pole. J Geophys Res doi: 10.1029/2004JD004937.

[5] Bernhard G, Booth CR, Ehramjian JC, Nichol SE (2006) UV climatology at McMurdo Station, Antarctica, based on version 2 data of the National Science Foundation’s Ultraviolet Radiation Monitoring Network. J Geophys Res doi: 10.1029/2005JD005857.

[6] Bernhard G, Booth CR, Ehramjian JC, Stone R, Dutton EG (2007) Ultraviolet and visible radiation at Barrow, Alaska: Climatology and influencing factors on the basis of version 2 National Science Foundation network data. J Geophys Res doi:10.1029/2006JD007865.

[7] Flemming J, Inness A, Jones L, Eskes HJ, Huijnen V, et al (2011) Forecasts and assimilation experiments of the Antarctic ozone hole 2008. Atmos Chem Phys 11: 1961–1977.

[8] Bernhard G, Booth CR, Ehramjian JC (2010) Climatology of Ultraviolet Radiation at High Latitudes Derived from Measurements of the National Science Foundation’s Ultraviolet Spectral Irradiance Monitoring Network. In: UV Radiation in Global Climate Change: Measurements, Modeling and Effects on Ecosystems, Gao W, Schmoldt DL, and Slusser JR editors, Springer-Verlag and Tsinghua University Press, ISBN: 978-3-642-03312-4.

[9] Wuttke S, Seckmeyer G, Koenig-Langlo G (2006) Measurements of spectral snow albedo at Neumayer, Antarctica. Annales Geophysicae 24: 7–21.

[10] Lenoble J (1998) Modelling of the influence of snow reflectance on ultraviolet irradiance for cloudless sky. Appl Opt 37: 2441–2447. 18273179 10.1364/ao.37.002441

[11] Pirazzini R (2004) Surface albedo measurements over Antarctic sites in summer. J Geophys Res doi: 10.1029/2004JD004617.

[12] Hall A (2004) The Role of Surface Albedo Feedback in Climate. J Climate 17: 1550–1568.

[13] Winton M (2006) Surface Albedo Feedback Estimates for the AR4 Climate Models. J Climate 19: 359–365.

[14] Vaughan DG, Marshall GJ, Connolley WM, King JC, Mulvaney R (2001) Devil in the Detail. Science 293: 1777–1779. 11546858 10.1126/science.1065116

[15] Aoki T, Kuchiki K, Niwano M, Kodama Y, Hosaka M, et al.. (2011) Physically based snow albedo model for calculating broadband albedos and the solar heating profile in snowpack for general circulation models. Journal of Geophysical Research doi: 10.1029/2010JD015507.

[16] Pedersen CA, Roeckner E, Lüthje M, Winther JG (2009) A new sea ice albedo scheme including melt ponds for ECHAM5 general circulation model. Journal of Geophysical Research doi: 10.1029/2008JD010440.

[17] Collins WD, Bitz CM, Blackmon ML, Bonan GB, Bretherton CS, et al (2006) The community climate system model version 3 (CCSM3). Journal of Climate 19 (11): 2122–2143.

[18] Grenfell TC, Warren SG, Mullen PC (1994) Reflection of solar radiation by the Antarctic snow surface at ultraviolet, visible, and near-infrared wavelengths. Journal of Geophysical Research doi: 10.1029/94JD01484.

[19] Grenfell TC, Perovich DK (2004) Seasonal and spatial evolution of albedo in a snow-ice-land-ocean environment. Journal of Geophysical Research Oceans doi: 10.1029/2003JC001866.

[20] Brandt RE, Warren SG, Worby AP, Grenfell TC (2005) Surface albedo of the Antarctic sea ice zone. Journal of Climate 18(17): 3606–3622.

[21] Meinander O, Kontu A, Lakkala K, Heikkilä A, Ylianttila L, et al (2008) Diurnal variations in the UV albedo of arctic snow. Atmos Chem Phys 8: 6551–6563.

[22] Meinander O, Kazadzis S, Arola A, Riihelä A, Räisänen P, et al (2013) Spectral albedo of seasonal snow during intensive melt period at Sodankylä, beyond the Arctic Circle. Atmos Chem Phys 13: 3793–3810.

[23] Cordero RR, Damiani A, Ferrer J, Rayas J, Jorquera J, et al (2013) Downwelling and Upwelling Radiance Distributions sampled under Cloudless Conditions in Antarctica, Applied Optics. 52(25): 6287–94. 10.1364/AO.52.006287 24085089

[24] Li S, Zhou X (2003) Assessment of the Accuracy of Snow Surface Direct Beam Spectral Albedo under a Variety of Overcast Skies Derived by a Reciprocal Approach through Radiative Transfer Simulation. Appl Opt 42: 5427–5441. 14526830 10.1364/ao.42.005427

[25] Meinander O, Wuttke S, Seckmeyer G, Kazadzis S, Lindfors A, et al (2009) Solar Zenith Angle Asymmetry Cases in Polar Snow UV Albedo. Geophysica 45(1–2): 183–198.

[26] Seckmeyer G, Bais A, Bernhard G, Blumthaler M, Booth CR, et al.. (2001) Part 1: Spectral instruments Instruments to Measure Solar Ultraviolet Radiation WMO-GAW No. 125 (Geneva, Switzerland: World Meteorological Organization).

[27] Wuttke S, Seckmeyer G, Bernhard G, Ehramjian J, McKenzie R, et al (2006) New spectroradiometers complying with the NDSC standards. J Atmos Ocean Technol 23(2): 241–251.

[28] Cede A, Herman J, Richter A, Krotkov N, Burrows J (2006) Measurements of nitrogen dioxide total column amounts using a Brewer double spectrophotometer in direct sun mode. J Geophys Res doi: 10.1029/2005JD006585.

[29] Gröbner J, Albold A, Blumthaler M, Cabot T, de la Casinière A, et al.. (2000) The variability of spectral solar ultraviolet irradiance in an Alpine environment. J Geophys Res doi: 10.1029/2000JD900395.

[30] Gröbner J, Blumthaler M, Kazadzis S, Bais A, Webb A, et al (2006) Quality assurance of spectral solar UV measurements: result from 25 UV monitoring sites in Europe, 2002 to 2004. Metrologia 43: S66–S71.

[31] Bais AF, Gardiner BG, Slaper H, Blumthaler M, Bernhard G, et al (2001) The SUSPEN intercomparison of ultraviolet spectroradiometers. J Geophys Res 106: 12509–12525.

[32] Cordero RR, Seckmeyer G, Pissulla D, DaSilva L, Labbe F (2008) Uncertainty Evaluation of Spectral UV Irradiance Measurements. Meas. Sci. Technol. 19: 1–15.

[33] Gröbner J, Hülsen G, Vuilleumier L, Blumthaler M, Vilaplana JM, et al.. (2007) Report of the PMOD/WRC-COST calibration and intercomparison of erythemal radiometers.

[34] Antón M, Serrano A, Cancillo ML, Vilaplana JM (2011) Quality assurance of broadband erythemal radiometers at the Extremadura UV Monitoring Network (Southwestern Spain). Atmospheric Research 100: 83–92.

[35] McKinlay AF, Diffey BL (1987) A reference action spectrum for ultraviolet induced erythema in human skin. Commission Internationale de l’Eclairage Journal 6: 17–22.

[36] Cordero RR, Seckmeyer G, Pissulla D, Labbe F (2008) Uncertainty of experimental integrals: application to the UV index calculation. Metrologia 45: 1–10.

[37] Balis D, Kroon M, Koukouli ME, Brinksma EJ, Labow G, et al.. (2007) Validation of Ozone Monitoring Instrument total ozone column measurements using Brewer and Dobson spectrophotometer ground-based observations. Journal of Geophysical Research doi: 10.1029/2007JD008796.

[38] McPeters R, Kroon M, Labow G, Brinksma E, Balis D, et al.. (2008) Validation of the Aura Ozone Monitoring Instrument total column ozone product. Journal of Geophysical Research doi: 10.1029/2007JD008802.

[39] Damiani A, De Simone S, Rafanelli C, Cordero RR, Laurenza M (2012) Three years of ground-based total ozone measurements in Arctic: comparison with OMI, GOME and SCIAMACHY satellite data. Remote Sensing of Environment 127: 162–180.

[40] Antón M, Cachorro VE, Vilaplana JM, Toledano C, Krotkov NA, et al (2010) Comparison of UV irradiances from Aura/Ozone Monitoring Instrument (OMI) with Brewer measurements at El Arenosillo (Spain) - Part 1: Analysis of parameter influence. Atmos Chem Phys 10: 5979–5989.

[41] Damiani A, Cabrera S, Muñoz RC, Cordero RR, Labbe F (2013) Satellite-derived UV irradiance for a region with complex morphology and meteorology: comparison against ground measurements in Santiago de Chile. International Journal of Remote Sensing doi: 10.1080/01431161.2013.796101.

[42] Douglass A, Fioletov V, Godin-Beekmann S, Müller R, Stolarski RS, et al.. (2011) Stratospheric ozone and surface ultraviolet radiation 1–80; Global Ozone Research and Monitoring Project-Report No. 52, 516 pp., Geneva, Switzerland.

[43] Tanskanen A, Manninen T (2007) Effective UV surface albedo of seasonally snow-covered lands. Atmos Chem Phys 7: 2759–2764.

[44] Cabrera S, Ipiña A, Damiani A, Cordero RR, Piacentini RD (2012) UV index values and trends in Santiago, Chile (33.5°S) based on ground and satellite data. Journal of Photochemistry and Photobiology B: Biology 115: 73–84. 10.1016/j.jphotobiol.2012.06.013 22883148

[45] Kazadzis S, Bais A, Balis D, Kouremeti N, Zempila M, et al (2009) Spatial and temporal UV irradiance and aerosol variability within the area of an OMI satellite pixel. Atmos Chem Phys 9: 4593–4601.

[46] Kazadzis S, Bais A, Arola A, Krotkov N, Kouremeti N, et al (2009) Ozone Monitoring Instrument spectral UV irradiance products: comparison with ground based measurements at an urban environment. Atmos Chem Phys 9: 585–594.

[47] Ialongo I, Buchard V, Brogniez C, Casale GR, Siani AM (2009) Aerosol Single Scattering Albedo retrieval in the UV range: an application to OMI satellite validation. Atmos Chem Phys Discuss 9: 19009–19033.

[48] Buchard V, Brogniez C, Auriol F, Bonnel B, Lenoble J, et al (2008) Comparison of OMI ozone and UV irradiance data with ground-based measurements at two French sites. Atmos Chem Phys 8: 4517–4528.

[49] Tanskanen A, Krotkov NA, Herman JR, Arola A (2006) Surface ultraviolet irradiance from OMI. IEEE transactions on Geoscience and Remote Sensing 44(5): 1267–1271.

[50] Tanskanen A, Lindfors A, Määttä A, Krotkov N, Herman J, et al.. (2007) Validation of daily erythemal doses from Ozone Monitoring Instrument with ground-based UV measurement data. J Geophys Res doi: 10.1029/2007JD008830.

[51] Weihs P, Blumthaler M, Rieder HE, Kreuter A, Simic S, et al (2008) Measurements of UV irradiance within the area of one satellite pixel. Atmos Chem Phys 8: 5615–5626.

[52] Ialongo I, Casale GR, Siani AM (2008) Comparison of total ozone and erythemal UV data from OMI with ground-based measurements at Rome station. Atmos Chem Phys 8: 3283–3289.

[53] Cordero RR, Seckmeyer G, Pissulla D, DaSilva L, Labbe F (2007) Uncertainty evaluation of the spectral UV irradiance evaluated by using the UVSPEC Radiative Transfer Model. Optics Communications 276: 44–53.

[54] Cordero RR, Seckmeyer G, Damiani A, Labbe F, Laroze D (2013) Monte Carlo-based Uncertainties of Surface UV Estimates from Models and from Spectroradiometers. Metrologia 50: L1–L5.

[55] Cordero RR, Damiani A, Laroze D, Dasilva L, Labbe F (2013) Spectral UV radiance measured at a coastal site: a case study. Photochemical & Photobiological Sciences 12: 1193. 23584370 10.1039/c3pp25440b

[56] Blumthaler M, Kreuter A, Webb A, Bais A, Kift R, et al. (2009) Albedo Effect on UV Irradiance, MOCA Joint Assembly, Montreal, Quebec, July 2009, Available: http://www.moca-09.org.Accessed 1 October 2013.

[57] Kylling A, Persen T, Mayer B, Svenøe T (2000) Determination of an effective spectral surface albedo from ground-based global and direct UV irradiance measurements. J Geophys Res 105(D4): 4949–4959.

[58] Smolskaia I, Masserot D, Lenoble J, Brogniez C, de la Casinière A (2003) Retrieval of the Ultraviolet Effective Snow Albedo During 1998 Winter Campaign in the French Alps. Appl Opt 42(9): 1583. 12665089 10.1364/ao.42.001583

[59] Badosa J, McKenzie RL, Kotkamp M, Calbó J, González JA, et al (2007) Towards closure between measured and modelled UV under clear skies at four diverse sites. Atmospheric Chemistry and Physics 7: 2817–2837.

[60] Satheesh SK, Srinivasan J, Vinoj V, Chandra S (2006) New Directions: How representative are aerosol radiative impact assessments? Atmospheric Environment 40(16): 3008–3010.

[61] Mayer B, Kylling A (2005) Technical note: The libRadtran software package for radiative transfer calculations - description and examples of use. Atmos Chem Phys 5: 1855–1877.

[62] Dahlback A, Stamnes K (1991) A new spherical model for computing the radiation field available for photolysis and heating at twilight. Planet Space Sci 39: 671–683.

[63] Gueymard CA (2004) The sun’s total and spectral irradiance for solar energy applications and solar radiation models. Solar Energy 76: 423–453.

[64] van den Broeke MR, Reijmer CH, van de Wal RSW (2004) A study of the surface mass balance in Dronning Maud Land, Antarctica, using automatic weather stations. Journal of Glaciology 50(171): 565–582.

[65] Parish TR, Bromwich DH (1987) The surface windfield over the Antarctic Ice Sheets. Nature 328: 51–54.

[66] Bromwich DH, Nicolas JP, Hines KM, Kay JE, Key E, et al.. (2012) Tropospheric Clouds in Antarctica. Rev Geophys doi: 10.1029/2011RG000363.

[67] Grooß JU, Brautzsch K, Pommrich R, Solomon S, Müller R (2011) Stratospheric ozone chemistry in the Antarctic: what determines the lowest ozone values reached and their recovery? Atmospheric Chemistry and Physics 11: 12217–12226.

[68] Stamnes K, Slusser J, Bowen M (1991) Derivation of Total Ozone Abundance and Cloud Effects from Spectral Irradiance Measurements. Applied Optics 30: 4418–4426. 20717220 10.1364/AO.30.004418

[69] Bernhard G, Booth CR, McPeters RD (2003) Calculation of total column ozone from global UV spectra at high latitudes. J Geophys Res doi: 10.1029/2003JD003450.

[70] Cordero RR, Seckmeyer G, Pissulla D, Labbe F (2009) Exploitation of Spectral Direct UV Irradiance Measurements. Metrologia 46: 19–25.

[71] Bais AF, Kazantzidis A, Kazadzis S, Balis DS, Zerefos CS, et al (2005) Deriving an effective aerosol single scattering albedo from spectral surface UV irradiance measurements. Atmos Environ 39(6): 1093–1102.

[72] Buchard V, Brogniez C, Auriol F, Bonnel B (2011) Aerosol single scattering albedo retrieved from ground-based measurements in the UV and visible region. Atmospheric Measurement Techniques 4: 1–7.

[73] Kleipool QL, Dobber MR, de Haan JF, Levelt PF (2008) Earth surface reflectance climatology from 3 years of OMI data. J Geophys Res doi: 10.1029/2008JD010290

[74] O’Byrne G, Martin RV, van Donkelaar A, Joiner J, Celarier EA (2010) Surface reflectivity from the Ozone Monitoring Instrument using the Moderate Resolution Imaging Spectroradiometer to eliminate clouds: Effects of snow on ultraviolet and visible trace gas retrievals. J Geophys Res doi: 10.1029/2009JD013079

